# Practical Thoughts on Tooth-Drawing

**Published:** 1854-07

**Authors:** C. T. Cushman


### *Practical thoughts on Tooth-Drawing.* By C. T. CUSHMAN, D. D. S.

### No. III.

"The various conditions of the teeth, which may create necessity for their extraction, produce equally great variety in the difficulty or ease with which the operation may be performed."—*Fox.*

"Though this operation may appear simple and easily performed, it however generally requires great experience, address, a correct knowledge of formation of the mouth, and the various peculiarities which are met with in the teeth, and their arrangement and diseases."—*Gariot.*

*Extraordinary difficulty in extracting a temporary tooth. Anomalous specimen.* CASE IX.—Master M——, at 4 years; nervo-lymphatic; was presented March 23d, 1852, to be relieved of a diseased tooth, the right superior lateral incisor.

Although he was not suffering acute pain from it, he complained of it occasionally, and his mother declared it to be so offensive that it was unpleasant to have him about her.

A glance at it showed that it was no common case. High up under the lip, and just over the apex of the root, was a flattened tumor of the gum, as large as a rifle-ball, hanging by a little pedicle.

On lifting this up with a probe, the end of the root could be seen through a small opening made in the socket by the discharging pus.

The tooth *was* offensive—so much so that a strong odor was imparted to the fingers in handling about it. Its history, so far as known, is interesting.

The crown was mostly destroyed by decay; but enough remained to show, that in width it was fully *twice the size of its mate.* The parents corroborated this, and had always considered it "a double tooth."

Why it should so early decay, may be regarded as singular, since the remaining ones were so generally sound. About one year previously, the child had had severe inflammation and swelling of the face, so as to quite disfigure the features, and nearly close the eyes. Neither the physician in attendance nor the parents suspected a *tooth* as the cause, until long after recovery, when the latter attributed it to this, which was doubtless the true source of the fever—originating in *periodontitis*.

The color of the remaining portion of the crown was of the peculiar light brown, which indicates disorganization, and total necrosis. Two distinct nerve canals could be traced with tha probe.

I apprehended no difficulty in extracting both roots (if united) with the forceps, and for this purpose took a small straight one, termed "infant forceps," applied it high up, but the substance crushed and compressed together like so much chalk. The next resource was to tap the roots for the screw. This I did with a conical burr-drill, and applied the screw.

The substance of the roots was likewise so completely softened (surely there is such a thing as *mollities dentium*, the effect of the dripping pus in this case)! it drew out the threads, both roots. I tried the punch—just so much would chip off as it embraced.

The screw forced deeper; the roots split. And now, although the sections were loose and movable, the difficulty was by no means at an end. They repeatedly eluded the grasp of a slim and delictae forceps, the extremities of which were filed in two bird-claw teeth, or when caught would chip off in minute pieces. But finally they were *fished* out; and thus was completed an operation in the outset seemingly simple but, as the sequel proved, quite formidable. The roots—they were evidently not united, although the crowns were—were tortuous, as is peculiar to most teeth of irregular or supernumerary growth.

A highly commendable degree of fortitude was exhibited by the little patient, worthy of maturer years.

I never was so baffled in a like case; never did I attempt such a peculiar substance.* The dentist who has never been foiled in this operation I should regard as a marvel. I know

* A year and a half afterward I removed a small section of the root showing the mark of the screw-threads. It had worked to the surface of the gum and was lying upon its side.

not how I could have better devised ; surely, no one *can* know how he would have *practically* succeeded in the case, with any different theoretical method.

*Superiority of the key over forceps in some cases, for extracting very firm-set teeth.* CASE X.—Mr. P——, aged about 30, temperament sanguino-nervous ; spare habit ; teeth generally sound, regular, and firm-set ; never had one extracted. Applied in great suffering from second left inferior molar. Tooth much decayed on buccal surface, but yet offered sufficient firmness for application of the forceps. He had four days previously given a dentist, a heavier man than myself, three fair trials on it, with the forceps. After exhausting himself, without loosening it in the slightest degree that I could perceive, he gave it up.

The patient had meantime suffered much pain from it, and was very anxious to be relieved ; but at the same time, as may be supposed, was in great excitement of apprehension about the result of further trials. I advised a trial with the key on first seeing the case, and gave considerable encouragement for hope of success. There was, however, one serious obstacle. The surrounding gum was so exceedingly inflamed and irritable he could not bear pressure even of the finger.

To obviate this difficulty I put the claw beyond the extremity of the key shaft—it has a sliding bolt, two mortices for the claw, also a screw in the end of the shaft—I padded the fulcrum and placed it outside, at the same time adjusting the claw on the tooth to be extracted. This brought the bolster exactly opposite the first molar where the gum would allow the necessary pressure. Thus, with one turn of no extraordinary force, and there is a considerable power lost with the claw so attached, I raised the tooth so that it was easily picked out with the forceps.

It was a formidable looking *grinder*, with long broad roots of that elliptical curve which renders them so strongly *attached* to their native cells. The man was delighted with my success ; and as to the pain of the operation, he said it was not to be compared to what he had previously undergone, to no effectual purpose, either in degree or duration.

*No. 2.—Illustrating the same. A rare pathological specimen.* Case XI.—Servant man, aged about 28; teeth large, white, and naturally clean; came to have his first left superior molar extracted. A dentist, also a heavier man than myself, had exerted his strength on it several times, three I believe, and given it up. It was as firm as ever.

I never felt more sincere sympathy for the ruin of a tooth than in this case. So large, so clean, and apparently so sound! However, a decayed opening was found in the crown, partly on the approximal and partly on the opposing surface. And *that* opening! It extended freely into the pulp-cavity, and I could look directly into the palatine root. It appeared full of red blood, and the surrounding gum attested a high degree of acute inflammation.

He had suffered *intensely* with it. But I was unwilling to extract such a noble-looking tooth. I probed the cavity and encouraged the bleeding; it bleed freely. Subsequently, for several days I applied every approved remedy I could think of to allay the inflammation, in the hope of plugging it finally, in some way, making it useful again and preventing the sacrifice of the tooth. But all in vain.

He returned again, and again, to be relieved *radically*. I applied the key. I extracted it with one effort only. No injury resulted to the socket, and no pain was complained of. Such roots! Three prongs triangularly diverging in a straight line from base to apex. The measurement was as follows: diameter of neck, $\frac{1}{2}$ inch; longest line drawn from apices of palatal and buccal roots, $\frac{3}{4}$ inch. The degree of inflammation

---

Note.—It might be inferred, from the two preceding cases, that I would recommend the key in preference to the forceps, for the extraction of the large molars. But I would not be so understood. I preferred the key because the forceps had previously failed in these cases, and I felt more sure of success with it. For general use I prefer forceps for all the large molars. The key is more *liable* to fracture the socket, though by no means invariably does so; while on the other hand, the forceps will sometimes unavoidably bring a small portion of the alveolus, although it be not embraced in the grasp. In this case it can only be detached with some difficulty. The key is *peculiarly* adapted to the easy and expeditious removal of the superior Bicuspides—the fulcrum placed on the inside.

may be inferred from the following fact: A large portion of the roots, and crown about the neck, is beautifully injected with bright arterial blood; making the tooth one of the most valuable and demonstrative specimens of the kind I have ever seen.

*Superiority of the key over forceps, for extracting a tooth that is deeply decayed on one side.* It will extract quicker. The operator making use of the sound side, need not prolong the operation, as he would necessarily do with the forceps, through well-grounded apprehension of crushing. With the forceps, if he succeeds at all, he thus inflicts much more pain, and, not unfrequently additional anguish by forcing one beak into the sensitive nervous pulp.

CASE XII.—*Ibid.* Aug. 20, 1851. Servant woman of Dr. B——, tooth the second right inferior molar. Applied the Harris forceps carefully to the neck—lingual side of the tooth crushed in without loosening. Substituted the key-bolster inside, extracted it instantly, with very little pain. This was certainly preferable to persisting in painful trials with the forceps for the sake of consistently saying, "I never use aught else." "I repudiate the barbarous turn-key," &c.

*The key for the superior bicuspides.*—I have said, for this class of teeth I prefer the key. I not only give myself more satisfaction in its use, but also to my patients, as they suffer less pain. The very form of the roots of these teeth, conical and wedge-shape, favors the use of the instrument, by offering an inclined plane for their lateral extraction.

Affording increased power, the operation is briefer, and consequently less painful. Using the forceps, I have occasionally been obliged to stop and rest after a "long pull," in extracting the superior bicuspides. With the key, not unfrequently do these teeth fall in my patient's lap. If the bolster, broad and flat, be padded with a fine napkin, its pressure on the gum is not complained of.

It is not always, however, that the key will succeed with these teeth. If the crowns are gone, the roots much hollowed out by decay, and brittle as a consequence, the screw is perhaps the only chance; and then after tapping, the root will not unfrequently split from its application. The thumb-fulcrum (hook,) will then generally bring out the palatal section, when the punch easily dislodges the remainder.

